# Evidence for Altered Canonical Wnt Signaling in the Trabecular Bone of Elderly Postmenopausal Women with Fragility Femoral Fracture

**DOI:** 10.1155/2016/8169614

**Published:** 2016-11-24

**Authors:** Simona Bolamperti, Isabella Villa, Alice Spinello, Greta Manfredini, Emanuela Mrak, Umberto Mezzadri, Marco Ometti, Gianfranco Fraschini, Francesca Guidobono, Alessandro Rubinacci

**Affiliations:** ^1^Bone Metabolism Unit, IRCCS Scientific Institute San Raffaele, Via Olgettina 60, 20132 Milano, Italy; ^2^Orthopaedic Unit, IRCCS Scientific Institute San Raffaele, Via Olgettina 60, 20132 Milano, Italy

## Abstract

Wnt signaling, a major regulator of bone formation and homeostasis, might be involved in the bone loss of osteoporotic patients and the consequent impaired response to fracture. Therefore we analyzed Wnt-related, osteogenic, and adipogenic genes in bone tissue of elderly postmenopausal women undergoing hip replacement for either femoral fracture or osteoarthritis. Bone specimens derived from the intertrochanteric region of the femurs of 25 women with fracture (F) and 29 with osteoarthritis without fracture (OA) were analyzed. Specific miRNAs were analyzed in bone and in matched blood samples. RUNX2, BGP, and OPG showed lower expression in F than in OA samples, while OSX, OPN, BSP, and RANKL were not different. Inhibitory genes of Wnt pathway were lower in F versus OA. *β*-Catenin protein levels were higher in F versus OA, whereas its cotranscriptional regulator (Lef1) was lower in F group. miR-204, which targets RUNX2, and miR-130a, which inhibits PPAR*γ*, were lower and higher, respectively, in F versus OA serum samples. The present study showed an inefficient Wnt signal transduction in F group despite higher *β*-catenin protein levels, consistent with the expected overall postfracture systemic activation towards osteogenesis. This transcriptional inefficiency could contribute to the osteoporotic bone fragility.

## 1. Introduction

Osteoporosis, a devastating and asymptomatic skeletal disorder of aging, characterized by compromised bone strength can result in bone fractures in response to minor trauma. Osteoarthritis is also a skeletal disorder affecting the elderly population, but, as several observational studies have shown, it provides protection to fragility fracture risk despite the age related loss of trabecular bone [1]. Microstructural analyses of the femoral neck have provided evidence for an adaptive mechanism of bone structure in osteoarthritis that might preserve the mechanical competence of bone. Indeed, it was shown that there is a different cortical and trabecular bone distribution in the femoral necks of osteoarthritis subjects when compared to the ones of osteoporotic postmenopausal women with femoral fracture [2]. The cortical and trabecular thickness was shown to be higher in osteoarthritis subjects even in the presence of low volumetric bone density [3, 4]. These structural differences, in particular the critical role of residual bone mass distribution, that is, cortical versus trabecular, better discriminate hip fracture likelihood than areal Bone Mineral Density (aBMD) by DXA [5]. As extensively discussed [6], several hierarchical levels of the femoral neck structure are altered in fracture cases due to the loss, during aging, of safety mechanisms that appear to be partially preserved in osteoarthritis. It is therefore critical to characterize the different biomolecular effectors in osteoporosis and osteoarthritis that lead to bone mass deterioration, especially the reasons for the loss of adaptive mechanisms in the former, in order to optimize the prevention and treatment of osteoporotic fractures. Several studies analyzing the expression of osteogenic genes in osteoporosis have been assigned to the Wnt pathway [7, 8]. The Wnt pathway is particularly relevant during mesenchymal stem cell (MSC) commitment towards osteoblastogenesis. When Wnt is active, the expression of the adipogenic transcriptions factors is inhibited, thus maintaining preadipocytes in an undifferentiated state [9]. Since the maintenance of bone integrity requires MSC commitment towards osteoblastic lineage [9], defective Wnt signaling in the osteoporotic bone could impair the tissue response to functional/mechanical demand and therefore increase the fracture risk. The downstream effector of Wnt activation, the transcriptional regulator *β*-catenin, plays disparate roles in different phases of bone remodeling and microdamage repair [10, 11] and might be the underlying critical factor determining the different bone mass distribution in osteoarthritis versus osteoporosis. Indeed, it is known that the mechanical adaptation of the skeleton is in part regulated by Wnt (see [12] for review) and that this mechanical adaptation appears to be better preserved in osteoarthritis versus osteoporosis [3].

Comprehensive analysis of several genes' expression has identified differences in both Wnt and transforming growth factor-*β*/bone morphogenetic protein pathways in bone of individuals with no evidence of joint disease (control) compared to individuals undergoing joint replacement surgery for either degenerative hip osteoarthritis (OA) or fractured neck of femur (F) [13]. All of these studies were important for the identification of the biomolecular targets that potentially play pathogenic roles in these skeletal disease processes. However, the ubiquitous loss of bone mass in the elderly, independent of the occurrence of OA or F, impairs full understanding of the causative roles of the different gene expression profiles observed.

Along this line of thought, we focused the present study on Wnt signaling to further characterize its efficiency in F patients compared to OA. We have therefore analyzed the expression of Wnt signaling genes and of Wnt modulated genes involved in osteoblastogenesis and adipogenesis in the bone tissue of postmenopausal women undergoing hip replacement for F or OA. Due to the emerging role of microRNAs (miRNAs) amongst the epigenetic factors involved in bone pathogenesis, which can further contribute to the modulation of Wnt signaling in bone remodeling [14–16], we evaluated the levels of selected miRNAs in bone and serum samples from these two groups of subjects.

## 2. Materials and Methods

### 2.1. Bone Samples

Trabecular bone samples from the intertrochanteric region of the femur at a site distal to the periarticular bone were obtained from 54 female patients undergoing hip replacement for femoral neck fracture (F,  *n* = 25; mean age: 82 ± 7 years) or for primary osteoarthritis (OA,  *n* = 29; mean age: 72 ± 10 years). In particular, trabecular bone was sampled from the distal side of metaphyseal cutting plane, required for the insertion of the femoral component [17, 18]. Bone and mineral metabolism parameters, such as PTH, 25(OH)D, Ca^2+^, and creatinine, were measured before surgery in both groups and bone mineral density (BMD) of vertebrae L1–L4, femur, and radius were measured before surgery for the OA group and at release from hospital for the F group (Table 1). None of the patients included in this study were taking any medication that affected bone or mineral metabolism. The study was approved by the Ethics Committee of the San Raffaele Scientific Institute (Protocol BMU-WNT, 25.03.2008), and the patients provided informed written consent.

### 2.2. Reverse Transcription and Semiquantitative Real-Time PCR

Bone specimens were rinsed thoroughly in cold PBS and cut into small pieces. Total RNA was extracted from minced bone biopsies using TRIzol™ reagent [13]. RNA (1 *μ*g) was reverse-transcribed using oligo (dT) primers (0.5 *μ*M), 200 U of M-MLV reverse transcriptase, deoxynucleotides (0.5 mM), 1x M-MLV reaction buffer, and RNasin ribonuclease inhibitor (1 U/*μ*L; Promega, Madison, WI, USA) in a total volume of 25 *μ*L. cDNA (10 *μ*g) was subjected to real-time PCR amplification using primer-probe sets validated and purchased as “Assay-on-Demand” from Applied Biosystems (Life Technologies Italia, Monza, Italy) in singleplex PCR mix. Real-time PCR reactions were performed in an ABI PRISM® 7900 Sequence Detection System (Life Technologies Italia, Monza, Italy). *β*-Actin was measured as housekeeping gene. Results are expressed according to the 2^−ΔCT^ method, where ΔCT = CT_gene_ − CT_housekeeping_.

### 2.3. Western Blot Analysis

Bone specimens were rinsed thoroughly in cold PBS, cut into small pieces, and incubated with rotation for 3 days in PBS-EDTA 14% at 4°C in order to decalcify the tissue. The pieces were then minced using an Ultra-Turrax in lysis buffer containing 50 mM Tris-HCl pH 7.4, 100 mM NaCl, 1% Triton, 5 mM EDTA, 1 mM DTT, and protease and phosphatase inhibitors. The lysates were centrifuged at 12,000 rpm for 10 min at 4°C, the supernatants were collected, and the total protein concentration was determined using the BCA assay (Pierce, Life Technologies Italia, Monza, Italy). Total protein extract (200 *μ*g) was mixed with an appropriate volume of denaturing Laemmli sample loading buffer, heated at 100°C for 5 min, and loaded onto 10% SDS polyacrylamide gels. Western blot analysis was performed using specific antibodies against human DKK1, *β*-catenin, GAPDH (Santa Cruz Biotechnology Inc., Heidelberg, Germany) and Lef1 (Cell Signaling Technology, Boston, MA, USA) diluted 1 : 200, 1 : 100, 1 : 1000 and 1 : 100, respectively, in 5% milk with 0.1% Tween 20. After washing, the membranes were treated with the appropriate horseradish peroxidase-conjugated secondary antibodies (1 : 2000). Bound peroxidase activity was revealed using enhanced chemiluminescence substrate (ECL, Pierce, Life Technologies Italia, Monza, Italy). Bands on X-ray films were then quantified using ImageJ 1.47v software (Wayne Rasband, NIH, USA), and the protein signals were normalized using the relevant GAPDH bands.

### 2.4. miRNA Assays

Bone tissue miRNAs were reverse-transcribed from total RNA using the TaqMan® MicroRNA Reverse Transcription kit (Life Technologies Italia, Monza, Italy). In accordance with the manufacturer's protocol (TaqMan Small RNA Assays, Life Technologies), for each RT reaction, 10 ng of RNA was combined with RT master mix containing 1 mM dNTPs, 50 U MultiScribe™ Reverse Transcriptase, 1x Reverse Transcription Buffer, 3.8 U RNase Inhibitor, and 1x of the kit-included specific primers. The reaction was incubated at 16°C for 30 min, at 42°C for 30 min, and then at 85°C for 5 minutes. From this reaction, 1.33 *μ*L was used for real-time PCR amplification. Real-time PCR was performed using purchased TaqMan microRNA Assay (Life Technologies Italia, Monza, Italy). The cDNA was mixed with 1x TaqMan microRNA primers and 1x TaqMan Universal PCR Master Mix II. The PCR reaction was performed using the same protocol as for the mRNAs.

Serum miRNAs were extracted using the miRNeasy Serum/Plasma kit (Qiagen S.p.A., Milano, Italy) according to the manufacturer's instructions. Retrotranscription and real time PCR were performed as described above. miRNA levels were quantified using the comparative threshold-cycle (CT) method, with snU6 used as the housekeeping gene, and the results were expressed according to the 2-ΔCT method, where ΔCT = CT_miRNA_ − CT_housekeeping_.

### 2.5. Statistical Analysis

Statistical analysis was performed with the statistical package Prism versus 4.00, (GraphPad Software, San Diego, CA, USA). Significance of the differences between groups was assessed by the Mann-Whitney test.

## 3. Results

Table 1 shows the reported age and the biochemical parameters of bone mineral metabolism of F and OA groups. The BMD of vertebrae L1–L4, neck and total femur, 1/3, and UD radius were not statistically different between the two groups. No significant differences were recorded in the levels of PTH, 25(OH)D, Ca^2+^, and creatinine, thus suggesting that the quality of bone architecture and/or other secondary factors is associated with bone fragility, independently of BMD [3].

### 3.1. Gene Expression

**Figure 1:**
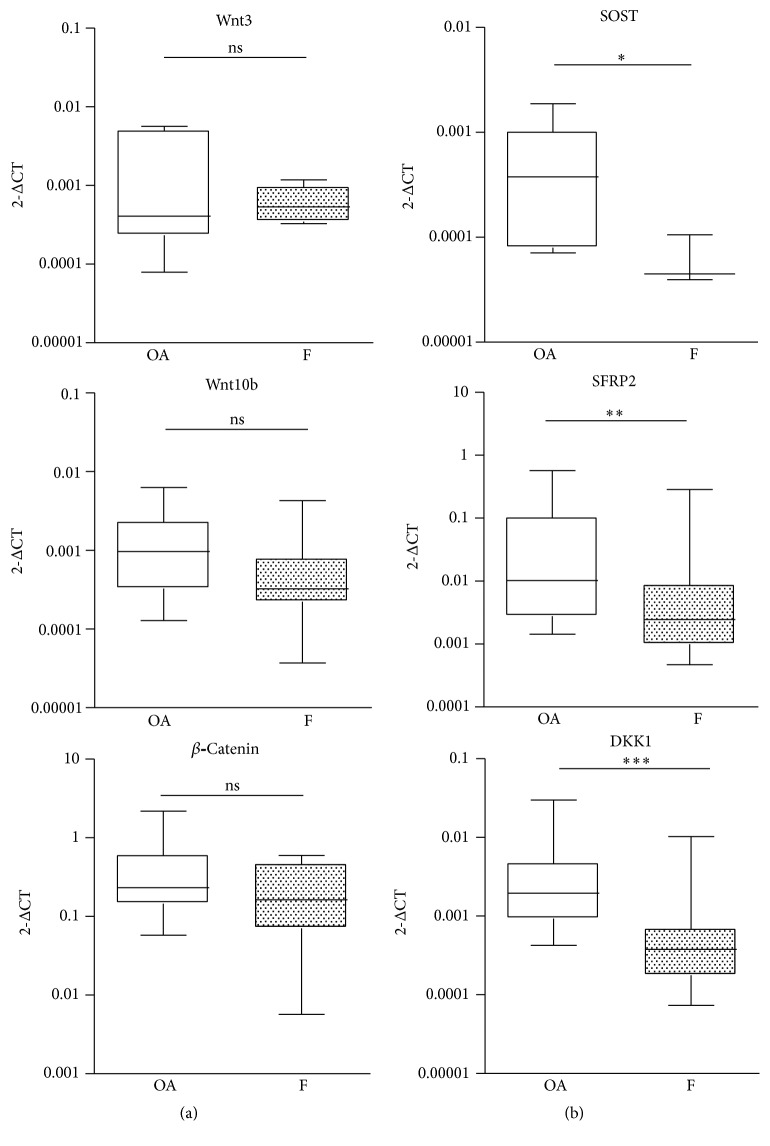
Gene expression of Wnt signaling activators (a) and Wnt signaling inhibitors (b) in bone samples of osteoarthritic (OA,  *n* = 29) and fractured (F,  *n* = 25) subjects. ^*∗*^ *p* < 0.05,  ^*∗∗*^ *p* < 0.01, and  ^*∗∗∗*^ *p* < 0.01, Mann-Whitney test.

**Figure 2:**
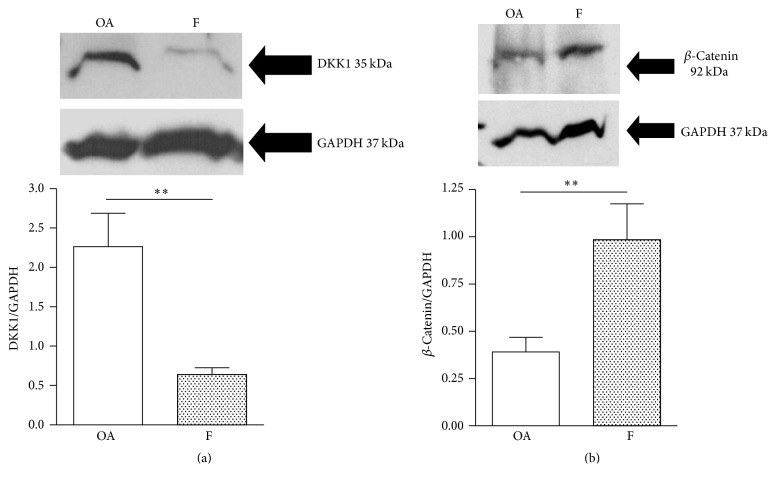
Representative western blot and relevant quantification of DKK1 protein levels (a) and of *β*-catenin protein levels (b) in bone samples of osteoarthritic (OA) and fractured (F) subjects (OA,  *n* = 4; F,  *n* = 5). ^*∗∗*^ *p* < 0.01, Mann-Whitney test.

The mRNA expression of Wnt-related genes was evaluated in F and OA groups. The expression of activators of canonical Wnt signaling, such as Wnt3 and Wnt10b, was comparable in the two groups, as was the expression of *β*-catenin, the transcriptional effector of the pathway (Figure 1(a)). Expression of inhibitors of Wnt signaling, such as SOST, SFRP2, and DKK1, was significantly lower (*p* < 0.05, *p* < 0.001, and *p* < 0.001, resp.) in the F group compared to the OA group (Figure 1(b)). Interestingly, SOST mRNA was detectable in the majority of OA subjects but, only in three of the F samples, it was undetected in the remaining samples due to a failure to reach the limit of detection in the PCR. In accordance with the PCR results, western blot analysis of DKK1 and *β*-catenin showed a lower (*p* < 0.01) and higher protein level (*p* < 0.01), respectively, in F samples (Figures 2(a) and 2(b)) compared with the OA group. The decrease in Wnt inhibitors and the consequent increase in *β*-catenin levels indicate that the canonical Wnt signaling pathway is highly activated in the F group.

**Figure 3:**
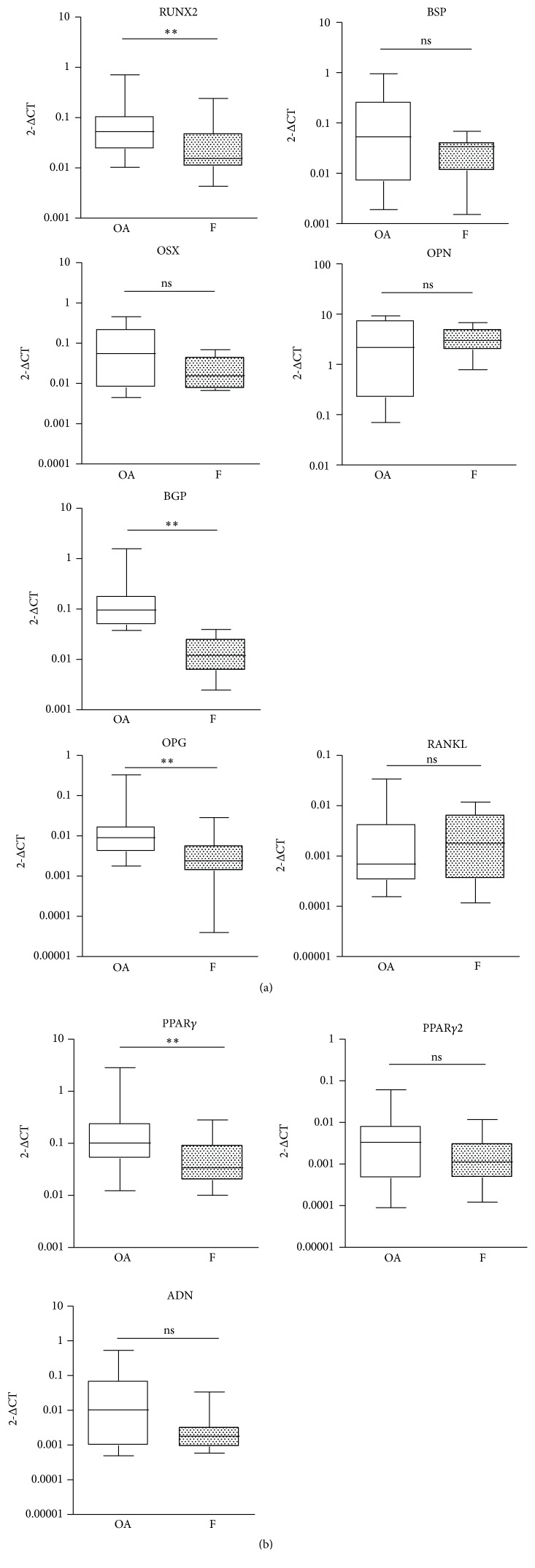
(a) Osteogenic gene expression in bone samples of osteoarthritic (OA,  *n* = 29) and fractured (F,  *n* = 25) subjects. (b) Adipogenic gene expression in bone samples of osteoarthritic (OA,  *n* = 29) and fractured subjects (F,  *n* = 25). ^*∗∗*^ *p* < 0.01, ns = not significant, Mann-Whitney test.

On the basis of these observations, we investigated the downstream effectors of active Wnt signaling. To do this, considering the role of Wnt in MSC differentiation, we evaluated the mRNA expression of genes involved in osteogenesis and adipogenesis. Despite the higher levels of *β*-catenin, real-time PCR results showed that osteogenic genes like RUNX2, BGP, and OPG had significantly lower expression (*p* < 0.01) in the F group compared to the OA group, whereas others like OSX, OPN, BSP, and RANKL displayed no significant difference between groups (Figure 3(a)). The comparable expression of RANKL between the two groups, along with a decreased OPG in the F group, suggests an environment favorable to bone resorption.

The evaluation of mRNA expression of adipogenesis-related genes showed that PPAR*γ*1 was significantly lower (*p* < 0.01) in the F compared to the OA group, whereas the expressions of PPAR*γ*2 and ADN (adiponectin) were not significantly different (Figure 3(b)). Given the similarity in expression of adipogenic genes between the groups (F versus OA), the observed decrease in genes related to osteogenesis in the F group suggests a possible enhanced adipogenesis/osteoblastogenesis ratio in the bone environment.

**Figure 4:**
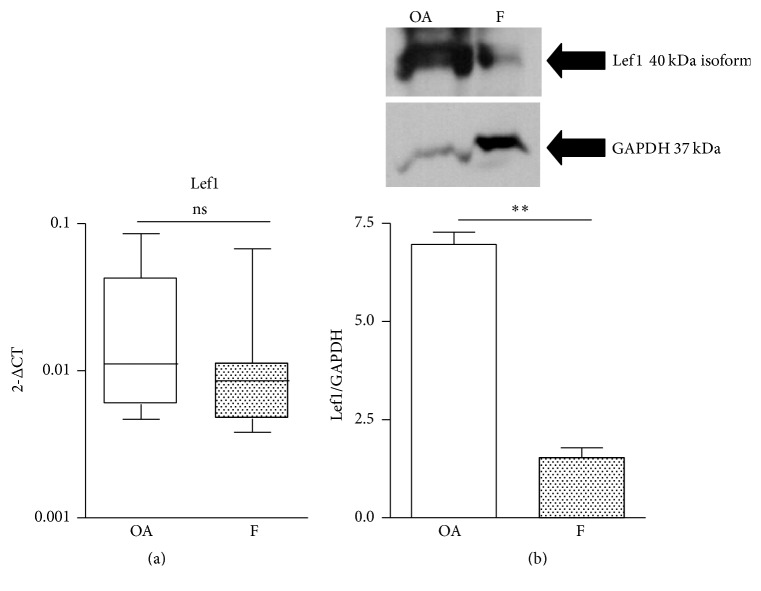
(a) mRNA expression of Lef1, in bone samples of osteoarthritic (OA, *n* = 10) and fractured (F,  *n* = 10) subjects. (b) Representative western blot and relevant quantification of Lef1 protein. ^*∗∗*^ *p* < 0.01, Mann-Whitney test.

The data so far indicated that in the F group Wnt was activated (high *β*-catenin) but could be transcriptionally inefficient (lower expressions of osteogenic genes). Considering that *β*-catenin regulates gene expression by binding transcription factors from the TCF/Lef1 family, we investigated the levels of Lef1. The mRNA expression of Lef1 showed a decrease in F versus OA but failed to reach statistical significance, probably due to the high variability in the OA group (Figure 4(a)). Nevertheless, the western blot results showed a significant (*p* < 0.01) decrease in Lef1 levels (Figure 4(b)). This reduction in Lef1 could be responsible, at least in part, for the observed inefficiency of Wnt signaling in the F group.

**Figure 5:**
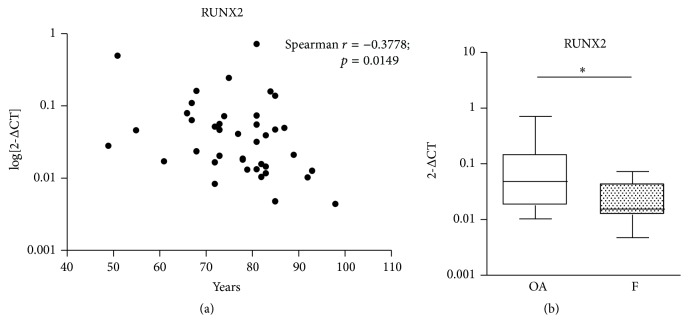
(a) Age related mRNA expression of RUNX2 in all the subjects included in the study. (b) RUNX2 mRNA expression in a subpopulation of age-matched subjects of the OA and F groups. ^*∗*^ *p* < 0.05, Mann-Whitney test.

The F group, as expected, showed a higher mean age than the OA group. To rule out any potential effect caused by the diverse age range, western blot comparisons were performed between age-matched samples and gene expression was tested for potential age-correlation by statistics. Only RUNX2 mRNA significantly correlated with age; that is, RUNX2 expression decreased with age (Spearman *r*: −0.378; *p* = 0.0149) (Figure 5(a)). Therefore, the statistical analysis on RUNX2 was performed again on age-matched subjects. This second analysis confirmed the observed lower expression of RUNX2 in F group compared to OA group (Figure 5(b)).

### 3.2. miRNA Expression

**Figure 6:**
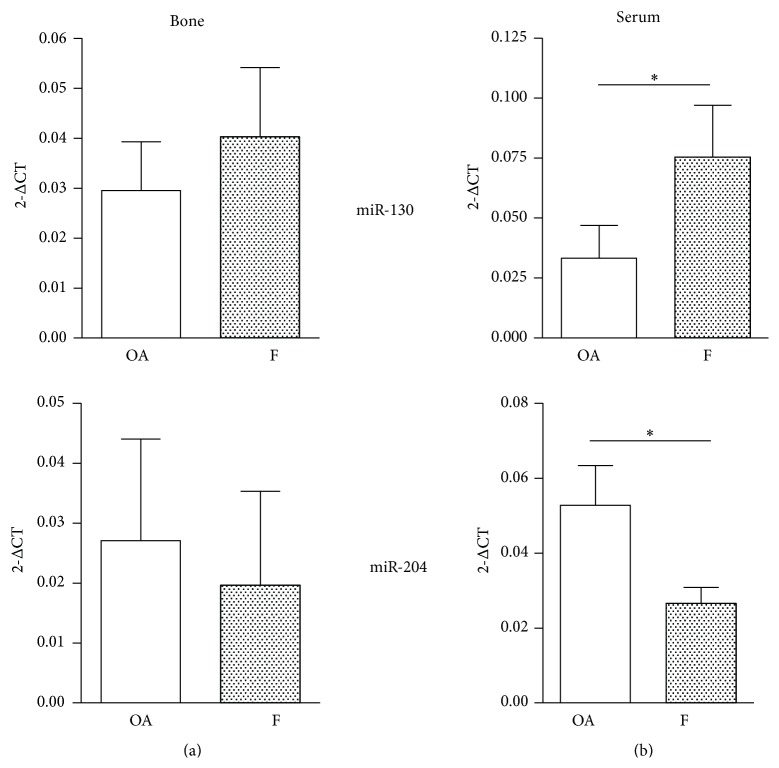
miRNA expression in bone samples (a) and sera (b) of osteoarthritic (OA, *n* = 17) and fractured (F,  *n* = 16) subjects. ^*∗*^ *p* < 0.05 Mann-Whitney test.

Based on the gene expression results, we next evaluated the expression of four miRNAs that are involved in the modulation of osteogenesis-related genes: miR-130, which inhibits PPAR*γ* mRNA expression [19]; miR-29a, which targets Wnt inhibitor expression [20]; miR-22, which targets both CDK6 and HDAC6, an antagonist of BMP2 signaling and a corepressor of RUNX2 [21], respectively; miR-204, which targets RUNX2 [22]. These miRNAs were measured in bone and serum samples from both the F and OA groups. The miR-130a level was higher in bone and serum samples from the F group, with the difference reaching statistical significance only in the serum samples (*p* < 0.05). The levels of miR-204 were lower in F group bone samples and reached statistical significance only in serum samples (*p* < 0.05, Figure 6). miR-29a and miR-22 levels were not significantly different in either bone or serum samples from the two groups (data not shown). These data, that is, increase of miR-130a and decrease of miR-204 in blood, suggest that upon fracture there is a skeletal response to facilitate bone healing. Since the same trend was observed in the bone specimens, it is likely that miRNAs were released by the skeleton directly into the blood stream to systemically circulate thus inducing a stronger and more effective osteogenic response by reaching distal skeletal sites.

## 4. Discussion

This study showed that the bone of a cohort of elderly postmenopausal women with fragility fracture of the femur (F) displays lower gene expression of Wnt signaling inhibitors and of osteogenesis-related genes compared to a similar cohort of postmenopausal women with osteoarthritis (OA) without fracture. The lower expression of DKK1, SFRP2, and SOST observed in the F cohort together with the increased protein levels of *β*-catenin did not lead to the activation of a Wnt-related osteogenic response. Indeed, the expressions of RUNX2, OSX, and BGP genes, which are target of the Wnt/*β*-catenin signaling pathway, were lower in F than OA. Our findings are consistent with previous observations showing that F and OA populations differ in their gene expression profiles [13] but provide additional information by showing that in the F cohort there is a lower expression of the Wnt-related osteogenic genes, despite a proven activation of Wnt signaling. In response to Wnt ligands, *β*-catenin translocates into the nucleus and binds a small family of DNA binding factors, the T cell factor/lymphoid enhancer factors (TCF/Lef). There are several TCFs factors but only one lymphoid enhancer factor, Lef1. When *β*-catenin binds TCF/Lef1, it can regulate the transcription of Wnt target genes [23]. The observed decrease in the protein amount of Lef1 in the F group in our study could be responsible, at least in part, for the reduced efficiency of Wnt signaling and the consequent reduced osteogenesis. In support of this hypothesis, female mice heterozygous for Lef1 (Lef1^+/−^) displayed low trabecular bone mass due to a reduced osteoblast activity [24].

Our study found that the F cohort, compared to OA, shows downregulated expression of RUNX2, a key transcription factor [25] that determines osteoblastic differentiation from mesenchymal precursors and is therefore required for the early stages of endochondral bone formation [26]. Low expression of RUNX2 might affect the downstream processes of osteoblast maturation [27]; thus, RUNX2 regulation might be critical in tipping the balance of bone remodeling away from equilibrium, with the result of bone fragility. Consistent with the observed lower expression of RUNX2, BGP expression was also lower in the F group compared to the OA group. BGP plays two major roles in human bone physiology in that it controls crystal morphology and it recruits osteoclasts [28]. Given its constitutive role in shaping the properties of bone matrix material [29], the lower expression of BGP observed in F versus OA patients could suggest that the bone of F patients is more brittle than that of OA patients. This view is in line with the higher fracture risk of an osteoporosis cohort compared to osteoarthritic individuals without fractures [4]. The difference in risk cannot be explained by differences in bone mass alone, as osteoarthritis does not seem to protect a patient from generalized primary osteoporosis [1]. Indeed, it has been shown that, contrary to common belief, these two disorders do coexist as osteoporosis does occur in a significant proportion of patients with end-stage osteoarthritis awaiting hip replacement [30]. The absence of any significant difference which has been found between bone density and mineral metabolism parameters in F versus OA cohorts is in line with this view and suggests that bone mass distribution could be critical for fracture risk. Our laboratory showed previously that the loading-induced adaptive distribution of residual bone mass, despite age and disease-related bone loss, is preserved in osteoarthritic subjects, accounting for their reduced fracture risk, while it is lacking in osteoporotic patients [3].

The strain signal does not appear to transduce an osteogenic response as RUNX2 levels were not increased in F samples compared to OA. SOST mRNA was only detectable in a few of the F samples most likely due to decreased mechanical competence, although we cannot exclude that the observed decrease is, like the other Wnt inhibitors, due to the active systemic response towards osteogenesis. The present study suggests that the defective adaptation mechanism and the deterioration of the trabecular network in osteoporosis [3] might be related, at least in part, to altered efficiency of the Wnt signaling.

We also observed reduced expression of OPG in the F group, providing additional evidence that accounts for bone fragility. Presumably, OPG expression, without any change in RANKL expression, may contribute to a shift in the remodeling balance towards increased resorption as seen in aging and osteoporosis. In agreement with the observation that osteoporotic bone with fragility fracture is characterized by an imbalance between osteoblastogenesis and adipogenesis [13], we found that the expression level of PPAR*γ*1 was lower in the F cohort, with no significant difference in PPAR*γ*2 levels. While PPAR*γ*1 is expressed by many cell types including osteoblasts and acts as inhibitor of Wnt signaling [31, 32], PPAR*γ*2 is expressed only by adipocytes [33] and has a dominant negative role in the regulation of osteoblastogenesis [34, 35]. It is therefore likely that the observed expression pattern, that is, unchanged expression of PPAR*γ*2 and adiponectin along with reduced expression of RUNX2, could yield a prevalent adipogenic environment in the fragile osteoporotic bone. This is in agreement with the observation that the adipogenesis/osteoblastogenesis ratio of precursor cells is enhanced in the bone marrow of osteoporotic subject [36].

Upon fracture there is a whole body response which supports osteogenesis through the delivery of systemic factors in the bloodstream [37]. Among such factors, there has recently been an emphasis on miRNAs, which are mainly studied for their role of biomarkers of a disease's state. In our study, the miRNAs circulating in the blood stream could also reflect their role in the healing process of the bone subjected to fracture. In serum samples from F versus OA the expression of miR-204, an endogenous negative regulator of RUNX2 that inhibits osteogenesis and promotes adipogenesis in mesenchymal progenitor cells [22] was lower in the F group than in the OA group. This supports the idea that there is a systemic promotion of osteogenesis upon fracture that is reflected locally by a decrease in Wnt inhibitors. In addition, the higher levels of miR-130a in sera samples from F group versus the OA group could contribute to the downregulation of PPAR*γ*1 in bone samples of F patients, in order to also promote osteogenesis. The clinical reports associating miRNA with bone pathologies are still limited and reviewed by Sun et al. [38]. The authors postulated that miRNA may play important role in bone remodeling and might be involved in the pathogenesis of osteoporosis. Accordingly, a recent study has shown a distinct circulating miRNA pattern in subjects with fragility fractures, suggesting that circulating miRNA could be a useful biochemical parameter to predict fracture risk [39].

This study was performed with human samples which provides a clinically translational aspect; however, it is not without limitations. The research did not include age-matched subjects without osteoarthritis and osteoporosis. Unfortunately, it is very challenging to find a healthy, age-matched control group, as previously discussed [3], since bone loss is a universal phenomenon linked to aging. The mean age of the subjects in this study was 75 years and the majority of postmenopausal women at this age are osteoporotic. Alternatively, postmortem matched bone samples from autopsies could be used; however, additional limitations include lack of circulating factors and poor RNA quality due to the lengthy time periods between death and sampling as mandated by Italian Legislation.

In this study, we have sourced the bone tissue from the intertrochanteric region where the prosthetic implant was going to be placed, in patients suffering both from fracture of the femoral neck and from osteoarthritis. The trabecular bone was sampled from the distal side of metaphyseal cutting plane, required for the insertion of the femoral component. Femoral head and neck were discarded. The intertrochanteric region, including calcar [17, 18], is an established sampling site for gene expression and histomorphometric analysis as it reflects the changes of bone microenvironment at a site away from the diseased joint surface in the OA and from the fracture gap in F.

In the F group the mean time interval between fracture and surgery was 3 days. It might be argued that during this time some inflammatory skeletal response, transiently activated by the first phase of fracture healing, might have acutely altered Wnt signaling pathway. However, as discussed by Logar et al. [40], samples were taken from the intertrochanteric region where the influences of the femoral neck fracture, if any, would be minimal. Although the intertrochanteric region is exposed to the overall response to the trauma, it is the only available source of bone tissue at the skeletal site critical for the osteoporosis outcome and that shows specific remodeling alteration in fractured individuals [3].

In conclusion, this study showed that soon after fracture has occurred there is a body systemic response to promote bone healing by activating osteogenesis [41]. This expected postfracture systemic response is suggested by a modification in sera of miRNAs favoring an osteogenic response of the bone and in a local decrease of Wnt inhibitors in bone that trigger *β*-catenin accumulation. However, although Wnt signaling was detectable up until the stabilization step of *β*-catenin, the final outcome, that is, upregulation of the gene expression of RUNX2 or OPG, did not occur. A reasonable explanation can in part be found in the lower levels of Lef1 in F group. In summary, impaired osteogenic response to mechanical demand in fragility-related femoral fracture in osteoporotic bone can be ascribed, at least in part, to an inefficient transduction of Wnt signaling and a coherent gene expression profile.

## Figures and Tables

**Table:** (a) Biochemical parameters and DXA measurements

	Age (years)	PTH (ng/mL)	25(OH)D (ng/mL)	Ca^++^ (mmol/L)	Crea mg/dL
OA	72 ± 12	84.9 ± 15	18.3 ± 4.4	1.15 ± 0.01	0.74 ± 0.05
F	82 ± 7^*∗∗∗*^	97 ± 10.2	19.4 ± 4.5	1.15 ± 0.01	0.87 ± 0.06

^*∗∗∗*^ *p* = 0.0005, Student's *t*-test.

**(b):** 

*T*-score		Vertebrae	Femur		Radius	
		L1–L4	Total	Neck	1/3	UD
OA	M ± SD range	−1.7 ± 1.3 (−3.0 → 0.2)	−1.8 ± 0.7 (−2.6 → −0.9)	−2.7 ± 1.3 (−5 → −1.3)	−2.6 ± 1.5 (−5 → −0.3)	−2.2 ± 1.2 (−3.7 → −0.2)

F	M ± SD range	−1.1 ± 1.6 (−3.4 → 0.7)	−2.2 ± 0.5 (−2.8 → −1.8)	−2.5 ± 0.7 (−3.4 → −1.6)	−2.9 ± 2.0 (−5 → 0.0)	−2.5 ± 1.5 (−4.2 → −0.7)

No statistical differences were detected between groups.

PTH: parathyroid hormone.

25(OH)D: 25 hydroxy vitamin D.

Ca^++^: ionized calcium.

Crea: creatinine.

UD: ultradistal.

## References

[1] Mäkinen T. J., Alm J. J., Laine H., Svedström E., Aro H. T. (2007). The incidence of osteopenia and osteoporosis in women with hip osteoarthritis scheduled for cementless total joint replacement. *Bone*.

[2] Blain H., Chavassieux P., Portero-Muzy N. (2008). Cortical and trabecular bone distribution in the femoral neck in osteoporosis and osteoarthritis. *Bone*.

[3] Rubinacci A., Tresoldi D., Scalco E. (2012). Comparative high-resolution pQCT analysis of femoral neck indicates different bone mass distribution in osteoporosis and osteoarthritis. *Osteoporosis International*.

[4] Vestergaard P., Rejnmark L., Mosekilde L. (2009). Osteoarthritis and risk of fractures. *Calcified Tissue International*.

[5] Yang L., Udall W. J. M., McCloskey E. V., Eastell R. (2014). Distribution of bone density and cortical thickness in the proximal femur and their association with hip fracture in postmenopausal women: a quantitative computed tomography study. *Osteoporosis International*.

[6] Reeve J., Loveridge N. (2014). The fragile elderly hip: mechanisms associated with age-related loss of strength and toughness. *Bone*.

[7] Hopwood B., Tsykin A., Findlay D. M., Fazzalari N. L. (2007). Microarray gene expression profiling of osteoarthritic bone suggests altered bone remodelling, WNT and transforming growth factor-*β*/bone morphogenic protein signalling. *Arthritis Research & Therapy*.

[8] Velasco J., Zarrabeitia M. T., Prieto J. R. (2010). Wnt pathway genes in osteoporosis and osteoarthritis: differential expression and genetic association study. *Osteoporosis International*.

[9] Ross S. E., Hemati N., Longo K. A. (2000). Inhibition of adipogenesis by Wnt signaling. *Science*.

[10] Chen Y., Whetstone H. C., Lin A. C. (2007). Beta-catenin signaling plays a disparate role in different phases of fracture repair: implications for therapy to improve bone healing. *PLoS Medicine*.

[11] Baht G. S., Silkstone D., Vi L. (2015). Exposure to a youthful circulation rejuvenates bone repair through modulation of *β*-catenin. *Nature Communications*.

[12] Manolagas S. C. (2014). Wnt signaling and osteoporosis. *Maturitas*.

[13] Hopwood B., Tsykin A., Findlay D. M., Fazzalari N. L. (2009). Gene expression profile of the bone microenvironment in human fragility fracture bone. *Bone*.

[14] Taipaleenmäki H., BjerreHokland L., Chen L., Kauppinen S., Kassem M. (2012). Mechanisms in endocrinology: micro-RNAs: targets for enhancing osteoblast differentiation and bone formation. *European Journal of Endocrinology*.

[15] Arfat Y., Xiao W.-Z., Ahmad M. (2015). Role of microRNAs in osteoblasts differentiation and bone disorders. *Current Medicinal Chemistry*.

[16] Seeliger C., Karpinski K., Haug A. T. (2014). Five freely circulating miRNAs and bone tissue miRNAs are associated with osteoporotic fractures. *Journal of Bone and Mineral Research*.

[17] Tsangari H., Findlay D. M., Zannettino A. C. W., Pan B., Kuliwaba J. S., Fazzalari N. L. (2006). Evidence for reduced bone formation surface relative to bone resorption surface in female femoral fragility fracture patients. *Bone*.

[18] Truong L.-H., Kuliwaba J. S., Tsangari H., Fazzalari N. L. (2006). Differential gene expression of bone anabolic factors and trabecular bone architectural changes in the proximal femoral shaft of primary hip osteoarthritis patients. *Arthritis Research & Therapy*.

[19] Lee E. K., Lee M. J., Abdelmohsen K. (2011). miR-130 suppresses adipogenesis by inhibiting peroxisome proliferator-activated receptor *γ* expression. *Molecular and Cellular Biology*.

[20] Kapinas K., Kessler C., Ricks T., Gronowicz G., Delany A. M. (2010). miR-29 modulates Wnt signaling in human osteoblasts through a positive feedback loop. *The Journal of Biological Chemistry*.

[21] Huang S., Wang S., Bian C. (2012). Upregulation of miR-22 promotes osteogenic differentiation and inhibits adipogenic differentiation of human adipose tissue-derived mesenchymal stem cells by repressing HDAC6 protein expression. *Stem Cells and Development*.

[22] Huang J., Zhao L., Xing L., Chen D. (2010). MicroRNA-204 regulates Runx2 protein expression and mesenchymal progenitor cell differentiation. *Stem Cells*.

[23] Archbold H. C., Yang Y. X., Chen L., Cadigan K. M. (2012). How do they do Wnt they do?: regulation of transcription by the Wnt/*β*-catenin pathway. *Acta Physiologica*.

[24] Noh T., Gabet Y., Cogan J. (2009). Lef1 haploinsufficient mice display a low turnover an low bone mass phenotype in a gender- and age- specific manner. *PLoS ONE*.

[25] Lian J. B., Stein G. S., Javed A. (2006). Networks and hubs for the transcriptional control of osteoblastogenesis. *Reviews in Endocrine and Metabolic Disorders*.

[26] McGee-Lawrence M. E., Carpio L. R., Bradley E. W. (2014). Runx2 is required for early stages of endochondral bone formation but delays final stages of bone repair in Axin2-deficient mice. *Bone*.

[27] Xiao G., Jiang D., Ge C. (2005). Cooperative interactions between activating transcription factor 4 and Runx2/Cbfa1 stimulate osteoblast-specific osteocalcin gene expression. *The Journal of Biological Chemistry*.

[28] Boskey A. L., Robey P. G., Rosen C. J. (2013). The composition of bone. *Primer on the Metabolic Bone Diseases and Disorders of Mineral Metabolism*.

[29] Poundarik A., Gundberg C., Vashishth D. (2011). Non-collageneous proteins influence bone mineral size, shape and orientation: a SAXS study. *Journal of Bone and Mineral Research*.

[30] Lingard E. A., Mitchell S. Y., Francis R. M. (2009). The prevalence of osteoporosis in patients with severe hip and knee osteoarthritis awaiting joint arthroplasty. *Age and Ageing*.

[31] Moldes M., Zuo Y., Morrison R. F. (2003). Peroxisome-proliferator-activated receptor *γ* suppresses Wnt/*β*-catenin signalling during adipogenesis. *Biochemical Journal*.

[32] Wan Y. (2010). PPAR*γ* in bone homeostasis. *Trends in Endocrinology and Metabolism*.

[33] Tontonoz P., Spiegelman B. M. (2008). Fat and beyond: The diverse biology of PPAR*γ*. *Annual Review of Biochemistry*.

[34] Moerman E. J., Teng K., Lipschitz D. A., Lecka-Czernik B. (2004). Aging activates adipogenic and suppresses osteogenic programs in mesenchymal marrow stroma/stem cells: the role of PPAR-*γ*2 transcription factor and TGF-*β*/BMP signaling pathways. *Aging Cell*.

[35] Kim M., Kim C., Choi Y. S., Kim M., Park C., Suh Y. (2012). Age-related alterations in mesenchymal stem cells related to shift in differentiation from osteogenic to adipogenic potential: implication to age-associated bone diseases and defects. *Mechanisms of Ageing and Development*.

[36] Justesen J., Stenderup K., Ebbesen E. N., Mosekilde L., Steiniche T., Kassem M. (2001). Adipocyte tissue volume in bone marrow is increased with aging and in patients with osteoporosis. *Biogerontology*.

[37] Kaspar D., Neidlinger-Wilke C., Holbein O., Claes L., Ignatius A. (2003). Mitogens are increased in the systemic circulation during bone callus healing. *Journal of Orthopaedic Research*.

[38] Sun M., Zhou X., Chen L. (2016). The regulatory roles of MicroRNAs in bone remodeling and perspectives as biomarkers in osteoporosis. *BioMed Research International*.

[39] Kocijan R., Muschitz C., Geiger E. (2016). Circulating microRNA signatures in patients with idiopathic and postmenopausal osteoporosis and fragility fractures. *Journal of Clinical Endocrinology & Metabolism*.

[40] Logar D. B., Komadina R., Preželj J., Ostanek B., Trošt Z., Marc J. (2007). Expression of bone resorption genes in osteoarthritis and in osteoporosis. *Journal of Bone and Mineral Metabolism*.

[41] Einhorn T. A., Gerstenfeld L. C. (2015). Fracture healing: mechanisms and interventions. *Nature Reviews Rheumatology*.

